# Precision Medicine in Vascular and Endovascular Surgery

**DOI:** 10.3390/jcm12031031

**Published:** 2023-01-29

**Authors:** Fadi Taher, Amun Hofmann, Afshin Assadian

**Affiliations:** Department of Vascular and Endovascular Surgery, Klinik Ottakring, 1160 Vienna, Austria

Personalized medicine and precision medicine are terms often used to refer to treatment strategies tailored specifically to individual characteristics of patients, as opposed to a *one-size fits all* approach. These characteristics pertain to differences among patients or subgroups of patients regarding their lifestyles or environments, but also, and potentially more importantly, to differences at a molecular or genetic level. Examples of such precision medicine strategies are abundant in the field of oncology, where diagnosis and treatment are frequently affected by specific patient and tumor characteristics. Over the last decade, the concept of personalized medicine has increasingly infiltrated cardiovascular medicine in general, as well as vascular surgery in specific.

Even before the popularization of the terms personalized medicine and precision medicine, it has not been unusual at all for decisions regarding a surgical treatment strategy to be made on a case-by-case basis. Albeit not necessarily at a molecular level, formulating a treatment plan for renal failure patients on how to achieve and maintain hemodialysis access, for instance, will consider environmental and patient-specific factors, the patient’s vascular status (including their outflow vein and arterial diameters and quality), as well as the patient’s own expectations and wishes. More recently, one could argue that patient-tailored approaches have been of increasing interest in vascular access care, where *fistula first* has been superseded by *the right access, the right patient, the right time*, and *for the right reason*. The investigation of molecular fingerprints and rise of high-throughput *omics* assays not only enhance our understanding of the life cycle of an arteriovenous fistula but enable the introduction of precision medicine principles for patients requiring renal replacement therapy [1]. 

Looking at aorto-iliac pathologies, recent advances in the realm of custom-made endografts have individualized the way we diagnose patients, plan their treatment, perform endovascular repair, and survey them afterwards. Reportedly, fenestrated endovascular aortic repair (FEVAR) using a custom-made device has increasingly been applied for pararenal aortic pathologies over the last decade [2] and may have all but replaced hybrid or endovascular repair using parallel grafts in the elective setting. The planning stage for this procedure involves the fabrication of a non-sterile endograft demo-device tailored to patient-specific anatomy that can be test-implanted in a three-dimensional aortic model of each individual patient [3]. This process to ensure a perfect or near-perfect fit of the device within the patient has recently been streamlined by omitting the demo-device and three-dimensional model in favor of a numerical simulation using finite element analysis of the patient’s aorta and the designed endograft [4].

Surveillance after endovascular aortic repair (EVAR), however, is still in many ways dominated by dogmatic principles. Considering the diverging evidence and plethora of potential imaging modalities for follow-up investigations, it appears reasonable to advocate patient-specific surveillance algorithms, taking into account cumulative radiation exposure, contraindications regarding pharmaceutical contrast enhancers, and center experience with different imaging technologies. B-Flow, a digitally encoded excitation ultrasound designed to optimize blood flow visualization [5], is currently under investigation regarding its ability to reliably detect endoleaks after EVAR without the need for contrast enhancement and has the potential to be a valuable technology for certain patients and centers. The treatment of such endoleaks after EVAR may also involve personalized strategies. FEVAR has been described as an option to treat type Ia [6] and type Ib endoleaks—the latter by implantation of an iliac fenestrated device (IFD) that may be feasible in anatomies that limit the use of off-the-shelf branched prostheses [7,8]. IFDs thereby may avoid a coil-and-cover approach which sacrifices blood flow via the internal iliac artery to achieve sufficient seal. The preservation of blood flow to the internal iliac arteries achievable by use of an IFD may avoid serious sequelae, such as gluteal claudication or even potentially debilitating complications such as colonic or spinal cord ischemia.

Preventing spinal cord ischemia in thoracoabdominal aortic repair has also seen impactful advances in the last several years and many concepts of precision medicine have been adopted. The treatment of thoracoabdominal aortic aneurysms will often involve the use of custom-made devices and will be specifically tailored to a patient’s anatomy. Different strategies to prevent, detect, and treat spinal cord ischemia may also be recommended for individual patients. These may include novel surveillance techniques, such as near-infrared spectroscopy [9], the use of spinal catheters, staging of the procedure, or minimally invasive segmental artery coil embolization [10].

While custom-made grafts may be used as an example for advances in endovascular surgery, as well as an area of vascular surgery that has adopted precision medicine concepts, the considered individual patient characteristics are largely anatomical. It appears likely that we will increasingly see personalized prediction or pharmacogenetic considerations in vascular surgery in light of patient-specific factors at the molecular, genetic, or even epigenetic level, with the ultimate goal to optimize vascular patient care.
